# Preface

**DOI:** 10.1007/s00281-020-00786-0

**Published:** 2020-03-11

**Authors:** Bianca Schaub

**Affiliations:** 1 University Children’s Hospital Munich, LMU Munich, Lindwurmstr.4, 80337 Munich, Germany; 2 German Lung Centre, CPC-M, Munich, Germany

In the current issue “*Asthma: Novel developments from bench to bedside*” a number of key topics relevant for asthma development reaching from epidemiology and lung function to the role of early immune regulation, genetics, and epigenetics as well as external factors such as viruses and the microbiome summarize recent knowledge paired with future perspectives for the next decade of asthma research and clinical translation. Insight into local airway immune responses translating from mice to men and the role of omics for asthma sum up the complex nature of this multifactorial disease. Several asthma phenotypes in combination with underlying endotypes are tackled in a number of the subsequent articles.

Asthma indeed represents a clinical syndrome affecting all age groups [1]. Asthma prevalence worldwide has rapidly increased in the last few decades. Although asthma prevalence has recently plateaued and even decreased in some areas of the world, continuing increase in other areas has been seen. Many risk factors and their distinct distribution in different areas of the world may explain the differences in prevalence, which will be discussed in “*Asthma epidemiology and risk factors*” in this issue [1]. In the article “*Lung functional development and asthma trajectories*” it is illustrated that a possible mechanism for a sustained effect of impaired lung function on asthma is the influence of early life risk factors on early life lung functional growth and development, representing the most susceptible phase of lung growth and plasticity. Hereditary and environmental peri- and postnatal factors on lung functional development are summarized including air pollution, tobacco exposure, nutrition, intrauterine growth retardation, prematurity, early life infections, microbiome, and allergies and their effect on lung functional trajectories [2]. While for example prematurity impairs lung growth directly, the influence of a number of environmental factors is mediated through inflammatory processes such as infections or oxidative stress. Timing and nature of these influences lead to degrees of impaired lung functional capacity in early adulthood [2]. Future long-term respiratory morbidity such as chronic asthma or chronic obstructive airway disease (COPD) is discussed as well as possibilities to prevent or modify early abnormal lung functional growth trajectories.

In parallel with lung development, it is critical how the immune system of a child responds to the interaction of genetic, epigenetic, and environmental factors and whether effective strategies for a balanced and healthy immune maturation can be assured to prevent asthma development [3]. Several phases of susceptibility in a child’s life are important. Pregnancy and early childhood are particularly susceptible for exogenous influences based on the developing nature of a child’s immune system. Endogenous influences such as family history and genetics are immutable, and epigenetic regulations can be modulated by both heredity and environmental exposures. Prenatal influences including mother’s nutrition, smoking, or infections influence the interplay of innate and adaptive immune regulation and peri- and postnatal influences including mode of delivery. Induction and continuous training of healthy maturation early in life comprise balanced innate immunity and an equilibrium of T cell subpopulations to counter-regulate pro-inflammatory or exuberant immune reactions. In later childhood, compensatory immune mechanisms are required to modulate deviant regulation of a child’s already primed immune trajectory [3]. The specific effects of exogenous and endogenous influences on a child’s maturing immune system are summarized in “*Role of early life immune regulation in asthma development*,” and it’s importance and potential intervention for early prevention and treatment strategies are delineated.

In addition to early life immune regulation, in “*Recent findings in the genetics and epigenetics of asthma and allergy*,” recent developments of a few key topics in this field are summarized. Several studies have focused on overlapping or specific phenotypes within the allergy spectrum and beyond, looking for common genetic traits shared between different diseases or disease entities. Importantly, asthma and allergy genetics in populations genetically different from European ancestry have been addressed, being key as the majority of new asthma patients are not white [4]. In epigenetics, several large-scale epigenome-wide association studies (EWAS) have been performed and interaction between environment and epigenetic signatures were of focus. Finally, the field of pharmacogenetics has been shown central to understand the susceptibility for and mechanisms of current asthma and allergy therapies, while in parallel, scientific answers to the availability of novel drugs promising individualized therapy are urgently needed [4].

While genetics and epigenetics are relevant for asthma development without doubt, respiratory viral infections are known as key triggers of asthma exacerbations. The common cold virus rhinovirus (RV) is the most prevalent pathogen circulating in the community with high diversity with approximately 170 genotypes, an effective replication in T helper 2 cell biased, inflammatory milieu, and specific risk genes. Decreased interferon responses, disrupted airway epithelial barrier, environmental exposures, and nutritional deficiencies increase the risk to RV and other virus infections [5]. The debate whether viruses are the hen (causative) or the egg in asthma development is ongoing. Time wise, respiratory syncytial virus (RSV) is the leading causative agent of bronchiolitis in the first year of life, whereas RV starts to dominate from the second year on. Breathing problems induced by either of these viruses is associated with subsequent asthma, with higher risk for children with severe RV-induced wheezing. RV mostly represents a risk factor for later atopic asthma, and RSV is more likely associated with subsequent non-atopic asthma. Treatments inhibiting inflammation (e.g., corticosteroids or anti-IgE) effectively decrease RV-induced wheezing and asthma exacerbations. The anti-RSV monoclonal antibody, palivizumab, decreases the risk of severe RSV illness and subsequent recurrent wheeze [5]. In “*Role of viruses in asthma*,” a better understanding of personal and environmental risk factors and inflammatory mechanisms leading to asthma is discussed central for developing new strategies for the prevention and treatment of asthma.

In the timely topic “*Dysbiosis of the gut and lung microbiome has a role in asthma*,” the authors describe how the development of asthma is influenced by environmental and exogenous factors synergizing with genetic predisposition, shaping the lung microbiome in particular during peri- and postnatal phases. A healthy lung microbiome is characterized by bacteria belonging to the phyla Bacteroidetes, Actinobacteria, and Firmicutes [6]. As one example for modulation, viral respiratory infections are associated with increased proteobacteria with genera *Haemophilus* and *Moraxella* in young children and adult asthmatics. This dysbiosis supports induction of inflammatory pathways contributing to bronchoconstriction and bronchial hyper responsiveness. Exogenous factors can influence the natural lung microbiota composition in a positive manner such as in farming environments or negatively such as allergens or air pollutants do. Gut microbiota dysbiosis also influences asthma pathogenesis. Antibiotics, antiulcer medications, and other drugs severely impair gut and lung microbiota. Resulting dysbiosis and reduced microbial diversity dysregulate the bidirectional crosstalk across the gut-lung axis, resulting in hypersensitivity and hyper reactivity to respiratory and food allergens. Efforts are undertaken to reconstitute the microbiota and immune balance by probiotics and engineered bacteria, but results from human studies do not yet support their efficacy in asthma prevention or treatment [6]. In summary, dysbiosis of gut and lung seem to be critical causes to the increased emergence of asthma.

In the article “*Modulating local airway immune responses to treat allergic asthma: lessons from experimental models and human studies*,” the current understanding of the contribution of local innate immune elements in the development and maintenance of inflammatory airway responses in mice and men is comprehensively summarized, and available leads for successful targeting of those pathways for future therapeutics are envisioned. Allergic asthma with Th2-type immunity represents 90% of child and 50% of adult asthma cases. Research on animal models of allergic disease has led to the generation of biologicals, a novel class of drugs, targeting components of Th2-type inflammation [7]. Although highly efficient in subclasses of patients, only symptomatic stages of diseases are targeted, and recently, it is suggested to rather focus on earlier stages in the inflammatory cascade as underlying cause of allergic airway inflammation. Thus, a focus on changing and redirecting the initiation of type 2 inflammatory responses against allergens and certain viral agents for example via innate immunity as driver of Th2-type immunity may be of longer-lasting, disease-modifying effects, if not even at some point cure for asthma [7].

Finally, “*Omics for the future in asthma*” focuses on refining of asthma phenotypes and understanding their underlying biological structure in order to facilitate precision medicine approaches. The use of a number of omics methods such as (epi)genomics, transcriptomics, proteomics, metabolomics, microbiomics, and exposomics are relevant to investigate asthma from diverse angles [8]. Although technological advancement led to increased application of omics studies in the asthma field, several design and methodological challenges still need to be tackled before omics can be truly translated to asthma patient care. Collaborations in centralized harmonized work frame such as consortia with consistent methodologies will support worldwide research teams to tackle these challenges. Challenges such as the lack of standardization of sampling and analytical methodologies and validation of findings need to be tackled for personalized patient care [8]. The authors envision an encouraging future of omics in asthma with a number of challenges for translation to patient care, which are reviewed.

Finally, this bouquet of further developing and novel fields of asthma research will only in concert be able to bring this complex field of this multifactorial disease forward. Thus, combined efforts across studies, country borders, and ethnicities are urgently needed. By harmonizing already available and designing novel well-defined cohorts together with clinically well-characterized phenotyping of the asthma syndrome, large, open consortia, well-prepared to integrate novel strategies of basic, clinical, and analysis methods, can offer answers to the currently unmet need, namely to understand distinct mechanisms of asthma endotypes and to treat the majority of asthma patients more individually and more efficiently. Finally, for asthma prevention, murine and human immunological studies in concert with genetic and epigenetic expertise are crucial to disentangle the key mechanisms for effective prevention strategies, early in life, but possibly also in childhood, later adolescence, and adulthood.
